# Immunohistochemical Features of MMP-9 and pSTAT1 in Granuloma Annulare and Sarcoidosis: A Comparative Study of 62 Cases

**DOI:** 10.1155/2023/4098459

**Published:** 2023-03-14

**Authors:** Ze Wu, Linghui Li, Hui Qu, Rui-Qun Qi, Jun Niu

**Affiliations:** ^1^Department of Dermatology, General Hospital of Northern Theater Command, No. 83 Wenhua Road, Shenhe District, 110016 Shenyang, China; ^2^College of Medicine and Biological Information Engineering, Northeastern University, No. 3-11, Wenhua Road, Heping District, 110819 Shenyang, China; ^3^Department of Dermatology, The First Hospital of China Medical University, No. 155 Nanjing Bei Street, Heping District, 110001 Shenyang, China; ^4^Key Laboratory of Immunodermatology, Ministry of Education and NHC, National Joint Engineering Research Center for Theranostics of Immunological Skin Diseases, No. 155 Nanjing Bei Street, Heping District, 110001 Shenyang, China

## Abstract

**Background:**

Granuloma annulare (GA) and sarcoidosis are granulomatous inflammatory diseases that share similarities.

**Objective:**

To identify the histological and immunohistochemical (IHC) features of GA and sarcoidosis.

**Methods:**

A retrospective review of 36 patients with GA and 26 with sarcoidosis was performed. Results from hematoxylin and eosin (H&E) staining and IHC staining of MMP-9 and pSTAT1 within the skin lesions of GA and sarcoidosis were analyzed, and random forest was applied for developing a predictive model.

**Results:**

Significantly greater expressions of MMP-9 (especially in elastic fibers, EFs, *P* < 0.0001) and pSTAT1 (*P* = 0.0003) were observed in lesion samples of GA versus sarcoidosis patients. In GA patients, MMP-9 was significantly upregulated in the interstitial type (*P* = 0.0222), while staining of pSTAT1 was positively correlated with the area of mucinous collagen in palisading GA (*R* = 0.5356, *P* = 0.0484). In sarcoidosis patients, MMP-9 (*R* = −0.7127, *P* = 0.0009) and pSTAT1 (*R* = −0.5604, *P* = 0.0067) were found to show stronger expressions in lesions with less lymphocyte infiltration. The predictive model demonstrated an AUC of 0.9675.

**Conclusion:**

These results indicate that MMP-9 and pSTAT1 might exert roles in granulomatous inflammation in different modes, and the presence of more robust MMP-9 staining in EFs appears to be more suggestive of GA.

## 1. Introduction

Granuloma annulare (GA) is a granulomatous skin disorder characterized by mucinous collagen degradation surrounded by palisading epithelioid histiocytes and lymphocytes [1]. Sarcoidosis is a multiorgan granulomatous disease, often involving the skin, with “classic” sarcoidosis showing “naked nodule” consisting of the aggregation of epithelioid histiocytes and scant lymphocytes [2]. However, the occurrence of clinical variants [3, 4] and atypical histological appearances pose diagnostic challenges when discriminating GA and sarcoidosis. For instance, interstitial GA characterized by histiocytes scattered in collagen bundles [4, 5], or a large amount of lymphocyte infiltration in sarcoidosis [6], can contribute to similarities between GA and sarcoidosis [7–9].

As a member of the matrix metalloproteinases (MMPs) family, MMP-9, which can be released from histiocytes in various granulomatous inflammatory diseases, can serve to assist in limiting the spread of bacteria such as Mycobacterium tuberculosis or other undetermined antigens. MMP-9 accomplishes these functions through the formation of well-organized granuloma [10–12], or by inducing molecules such as transforming growth factors-*β* (TGF-*β*), which can further mediate fibrosis [13]. However, it has been also reported that MMPs can participate in bacterial dissemination via extracellular matrix degradation [12].

Interactions between macrophages and T cells are believed to be involved with the development of GA and sarcoidosis. This follows that as interferon-*γ* (IFN-*γ*) secreted by CD4+ T cells leads to the signal transducer and activator of transcription 1 (STAT1) activation in macrophages; then, the polarized macrophages produce interleukin-6 (IL-6) to activate T cells in turn; this loop maintains the persistence of granulomatous inflammation [14–16]. In granulomatous diseases, M1 macrophages (classically activated) promote T helper 1 (Th1) response and microbicidal activity, while M2 macrophages (alternatively activated) play roles in tissue repair and regeneration [17]. The balance between M1 and M2 macrophages in various diseases are, in part, regulated by STAT1/3 [18]. With regard to GA and sarcoidosis, the Janus kinase- (JAK-) STAT pathway has been identified as a target in the treatment of these two conditions [14, 15, 19]. Moreover, JAK-STAT signaling correlates to the severity of sarcoidosis [20].

Herein, we aim to compare the histological features and investigate the expression of MMP-9 and pSTAT1 between GA and sarcoidosis.

## 2. Materials and Methods

### 2.1. Patients

A retrospective study was performed by searching the electronic pathology database for the terms “granuloma annulare” and “sarcoidosis” from 2002 through 2019. The retrieved 39 cases of GA and 108 cases of sarcoidosis were further determined as 36 and 26 by excluding cases with unclear diagnosis or insufficient tissue left in the wax block, which reviewed by professional dermatologists and pathologists based on the combination of clinical and histological data. We collected demographic and clinical data from patient files.

### 2.2. Tissue Microarray (TMA)

The formalin-fixed and paraffin-embedded (FFPE) samples of GA and sarcoidosis were collected from the dermatopathological department of General Hospital of Northern Theater Command. Tissue cores of 1.5 mm in diameter were drilled from selected donor blocks at the position with typical histopathological features of granuloma evaluated by pathologist from hematoxylin and eosin (H&E) staining slides. Press 62 separate tissue cores into the recipient block for TMA.

### 2.3. Immunohistochemistry (IHC)

Deparaffinization and hydration with absolute ethanol were performed at the 4-micron TMA slides; then, heat-mediated antigen retrieval was performed in tris-ethylene diamine tetraacetic acid buffer (pH 9.0, 97°C, 10 min). After antigen blocking, the slides were incubated with a rabbit monoclonal MMP-9 antibody (Abcam, UK, ab76003) and a rabbit monoclonal phospho-STAT1 antibody (Abcam, UK, ab109461) at 4°C overnight. Afterward, horseradish peroxidase (HRP) conjugated goat antirabbit secondary antibody (Abcam, UK, ab205718) was applied for 30 minutes at 37°C. After washing, slides were incubated with diaminobenzidine (DAB).

### 2.4. Histopathologic and Immunohistochemical Data

For the TMA sections, Vectra system (Akoya Biosciences, DE, USA) was run to get the spectral information. The image files were then analyzed to get cell density, selected tissue category area, and optical density information of MMP-9 and pSTAT1 using inForm 2.4 image analysis software (Akoya Biosciences, DE, USA). We accessed the expression of MMP-9 and pSTAT1 of all components in one selected tissue category using (1) IHC score, which evaluate the intensity and percentages of components with staining above threshold set for strong, medium, and weak staining or (2) positivity, showing the percentages above thresholds, while using (3) IOD/area, the mean integrated optical density (IOD) per distribution area to qualified the expression MMP-9 and pSTAT1 of selected component. H&E staining section of biopsy tissue from each patient was examined for area of granuloma, depth of inflammation, and area of mucinous collagen using the NanoZoomer HT Scan system 2.0 (Hamamatsu, Japan).

### 2.5. Predictive Model

*T*-test was used in order to screen out the features with significant difference between GA and sarcoidosis; then, random forest (RF) model was performed based on leave-one-out cross-validation (LOOCV). The least absolute shrinkage and selection operator (LASSO) algorithm was used for feature dimension reduction before each training.

### 2.6. Statistical Analysis

Continuous variables were compared using Student's *t*-test. Chi-square test or Fisher's exact test was used to compare the categorical variables. The correlation was evaluated by Pearson correlation test. Precision, recall, and *F*1-score were calculated for model performance evaluation. Receiver operating characteristics (ROC) curves were used to evaluate the diagnostic efficiency. SPSS 26.0 and GraphPad Prism 7.4 software were used for statistical analysis and graph plotting. Generally, *P* values less than 0.05 were considered statistically significant.

## 3. Results

### 3.1. Patient Characteristics

**Figure 1:**
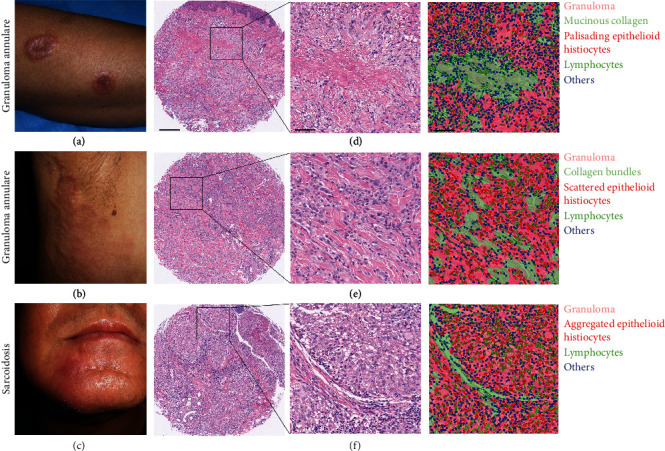
Granuloma annulare and sarcoidosis. (a, b) Clinical photographs for granuloma annulare (GA). (c) Clinical photograph for sarcoidosis. (d) Hematoxylin and eosin (H&E) staining and segmentation of GA biopsy section with palisading pattern of infiltrate. (e) H&E staining and segmentation of GA biopsy section with interstitial pattern of infiltrate. (f) H&E staining and segmentation of sarcoidosis biopsy section. Lower-power photo, scale bar = 250 *μ*m; high-power photo, scale bar = 60 *μ*m.

36 patients with GA (Figures 1(a) and 1(b)) and 26 with sarcoidosis (Figure 1(c)) were included in the study. There were more male patients in GA than in sarcoidosis (GA 88.5%, sarcoidosis 34.6%; *P* = 0.023). The median age at diagnosis in GA was younger than that in sarcoidosis (GA 47y, sarcoidosis 53y; *P* = 0.019). Upper limbs were the most frequently involved anatomic sites (41.7%) for GA, while upper limbs (53.8%) and head (53.8%) for sarcoidosis (Table 1). Histologically, of the 36 GA cases reviewed, 36.1% showed palisading pattern (Figure 1(d)), while 63.9% showed an interstitial granuloma (Figure 1(e)). There were no significantly fewer lymphocytes in sarcoidosis than that in GA (GA 228.5, sarcoidosis 257.1; *P* = 0.45) even if sarcoidosis is well known as the “naked nodule” with limited lymphocytes infiltrate (Figure 1(f)). When compared with GA, deeper median granulomatous inflammatory infiltration (GA 1360.0 *μ*m, sarcoidosis 3140.0 *μ*m; *P* < 0.01) and larger median granulomatous area (GA 2.5 mm^2^, sarcoidosis 6.5 mm^2^; *P* < 0.01) were observed in sarcoidosis. (Supplemental Figure 1A-1C).

### 3.2. MMP-9 Distinguishes GA from Sarcoidosis

**Figure 2:**
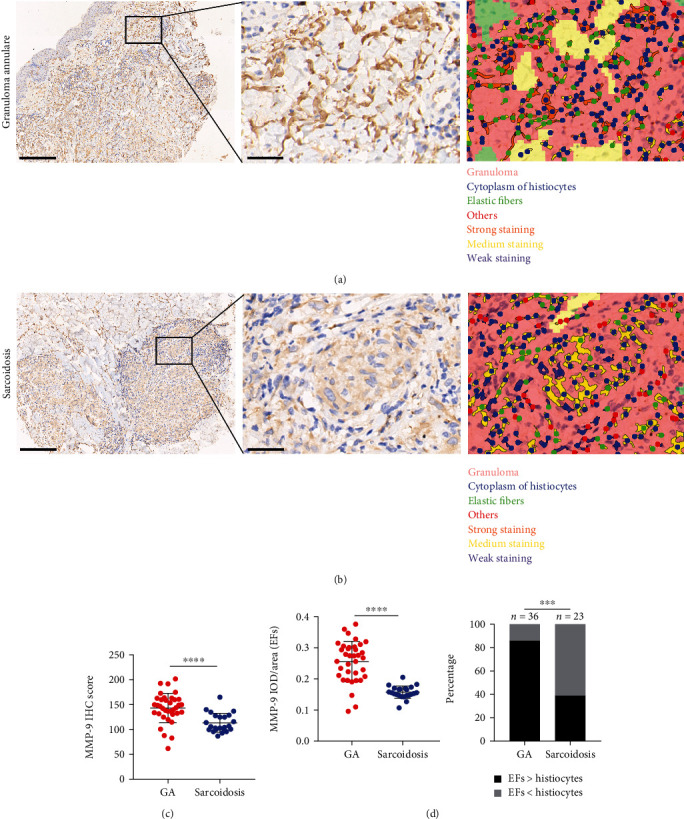
MMP-9 expression in granuloma annulare and sarcoidosis biopsies. (a) IHC staining and segmentation of biopsy section of patients with GA, the high-power photo shows that MMP-9 predominantly stain the elastic fibers (EFs) in GA biopsy. (b) IHC staining and segmentation of biopsy section of patients with sarcoidosis, the high-power photo shows that MMP-9 predominantly stains the histiocytes in sarcoidosis biopsy. Lower-power photo, scale bar = 250 *μ*m; high-power photo, scale bar = 40 *μ*m. (c) Quantification of IHC staining for MMP-9 in patients with GA and sarcoidosis. (d) Quantification of IHC staining for MMP-9 in EFs in patients with GA and sarcoidosis. MMP-9 is mainly expressed in EFs in GA while histiocytes in sarcoidosis.

We found that the intensity of MMP-9 staining in GA samples was stronger than that in sarcoidosis (Figures 2(a)–2(c)). Although MMP-9 was expressed in both histiocytes and elastic fibers in both diseases (quantified using IHC score or positivity), significant differences in MMP-9 staining within elastic fibers (quantified using IOD/Area) were present, with more robust staining in the thin, wavy, and fragment appearance elastic fibers (Figure 2(a), middle column) being observed in the GA group (Figure 2(d)). This trend was also present when the analysis was confined to that of male patients and patients over 50 years of age. However, no statistically significant differences in MMP-9 staining were present between the two groups with regard to lesions on limbs or lesions of patients with disease durations greater than three years (Supplemental Figure 2A).

### 3.3. MMP-9 in GA and Sarcoidosis

**Figure 3:**
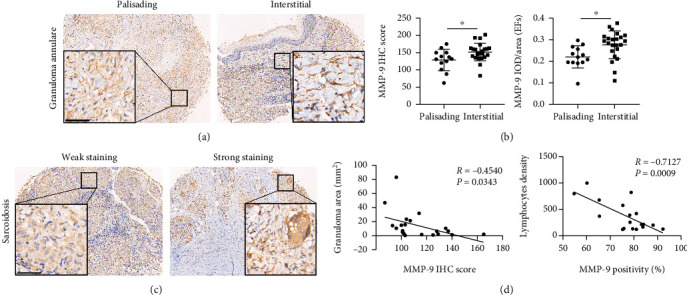
MMP-9 expression in granuloma annulare and sarcoidosis biopsies. (a) IHC staining of MMP-9 in palisading (left panel) and interstitial (right panel) GA, scale bar = 55 *μ*m. (b) Quantification of IHC staining for MMP-9 in patients with palisading and interstitial GA. (c) IHC of MMP-9 in sarcoidosis patients with weak (left panel) and MMP-9 strong (right panel) staining, scale bar = 55 *μ*m. (d) Correlation between MMP-9 expression and granuloma area and lymphocyte density in sarcoidosis.

MMP-9 staining in interstitial GA was significantly stronger compared with that observed in palisading GA, with this staining being predominantly expressed in elastic fibers (Figures 3(a) and 3(b)). In those patients with palisading granuloma as their main histological appearance, a larger granuloma was present in the dermis, and these patients showed a longer course of disease duration (Supplemental Figure 1D-1F). The percent of palisading granuloma in annular lesions was approximately equal to that in nonannular lesions (data not shown).

The clinical manifestation of sarcoidosis varies, notably, we found that deeper inflammatory infiltration and more multinucleated giant cells (MNGCs) in the nodular lesions (Supplemental Figure 1G-1I). Moreover, stronger MMP-9 staining was found in sarcoidosis biopsies, along with smaller granulomas and less lymphocyte infiltration (Figures 3(c) and 3(d)).

### 3.4. Different Staining of pSTAT1 between GA and Sarcoidosis

**Figure 4:**
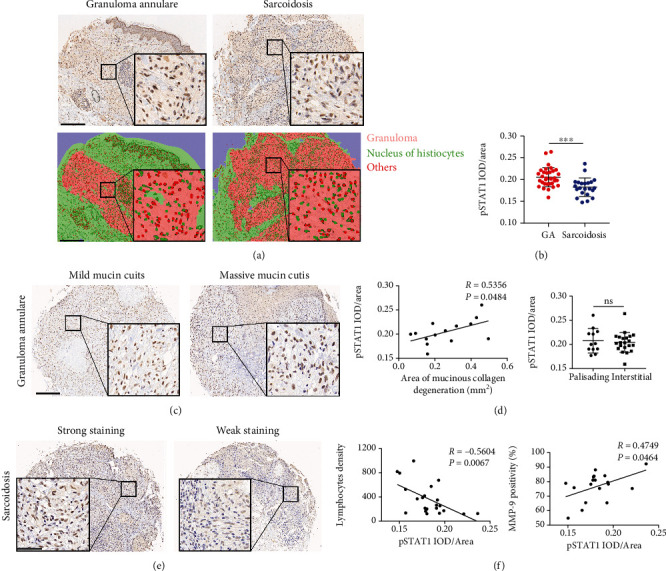
pSTAT1 expression in granuloma annulare and sarcoidosis biopsies. (a) IHC staining (upper row) and segmentation (lower row) in patients with GA (left column) and sarcoidosis (right column), scale bar = 250 *μ*m. (b) Quantification of IHC staining for pSTAT1 in patients with GA and sarcoidosis. (c) pSTAT1 IHC in GA patients with mild (left panel) and massive (right panel) mucin cutis and collagen denaturation, scale bar = 250 *μ*m. (d) Correlation between pSTAT1 expression and area of mucin cutis and collagen denaturation in GA; quantification of IHC staining for pSTAT1 in palisading and interstitial GA biopsies. (e) pSTAT1 IHC in sarcoidosis patients with pSTAT1 strong (left panel) and weak (right panel) staining, scale bar = 55 *μ*m. (f) Correlation between pSTAT1 expression and lymphocyte density; correlation between pSTAT1 and MMP-9 expression in sarcoidosis.

Activated STAT1 (phosphorylated) was nuclear positive in histiocytes in both diseases (quantified using IOD/area), but stronger in GA biopsies (Figures 4(a) and 4(b)), both in male and female subsets, pSTAT1 staining was increased in GA lesions in patients older than 50 years, but minimal differences between the two diseases were observed in young and middle-aged subjects. There were statistically significant increases in levels of pSTAT1 expression in the GA lesions compared with sarcoidosis in the trunk and limbs lesions, but not in lesions located in the head and neck. As the course of these diseases progressed, levels of pSTAT1 staining tended to be similar in the two diseases (Supplemental Figure 2B).

### 3.5. pSTAT1 in GA and Sarcoidosis

In GA, the staining intensity of pSTAT1 in histiocytes was positively correlated with the area of mucinous collagen degeneration, while there was no difference in pSTAT1 staining between palisading and interstitial GA (Figures 4(c) and 4(d)).

In sarcoidosis, with the increase of pSTAT1 staining in the nucleus of histiocytes, there was a significant decrease in lymphocytes infiltration. Moreover, a positive correlation between MMP-9 expression and pSTAT1 expression was noted (Figures 4(e) and 4(f)).

### 3.6. Predictive Model

**Figure 5:**
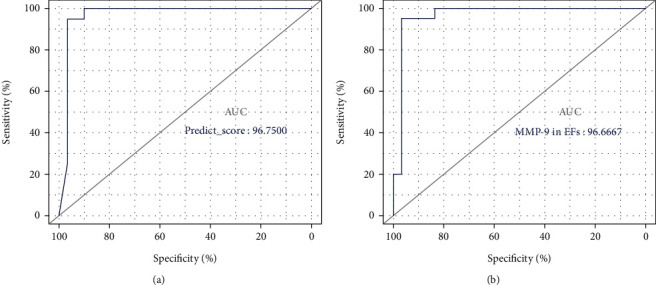
ROC curves of predictive model and variable MMP-9 in elastic fibers. (a) The RF predictive model based on 5 variables as sex, age, deepness of granulomatous infiltration, MMP-9 IOD/area in elastic fibers, and pSTAT1 IOD/area in the nuclei of histiocytes showed an AUC of 0.9675. (b) MMP-9 in elastic fibers obtained an AUC of 0.9667.

Five variables, including sex, age, depth of granulomatous infiltration, MMP-9 IOD/area in EFs, and pSTAT1 IOD/area demonstrated importance; the random forest model had 0.944 precision, 0.850 recall, and 0.895 *F*1-score and showed an AUC of 0.9675 (Figure 5(a)). A highest AUC of 0.9667 was obtained with variable MMP-9 staining in EFs (Figure 5(b)).

## 4. Discussion

The formation of granulomas involves macrophage activation and accumulation in tissue [15, 16]. In our study, we examined the expression of pSTAT1, a molecule expressed in macrophages to respond to lymphocytes. Previous studies have demonstrated the contribution of gelatinase MMP-2 and MMP-9 in cell migration [21], and we explored the role of MMP-9 in macrophage recruitment in GA and sarcoidosis.

Granuloma annulare is an inflammatory skin disease. This condition is not considered to be a unique disease but rather a reaction to a variety of factors such as malignancy, trauma, thyroid disease, diabetes mellitus, and viral infection [22]. The pathogenesis of GA may involve a Th1 cell-mediated delayed-type hypersensitivity reaction to an unknown antigen [22, 23]. T cells, macrophages, and fibroblasts are found in the tissue microenvironment of GA skin lesions [15]. When these recruited macrophages become activated, they release proinflammatory cytokines such as matrix metalloproteinases leading to collagen destruction and tissue remodeling [24]. Moreover, high expression levels of MMP-9 near caseous necrosis can promote tuberculosis progression by destroying the granulomatous structure [25].

In this study, the GA lesion samples showed typical histological features, including collagen denaturation, histiocyte infiltration, and mucin deposition [1]. Based on these characteristics, it seems reasonable to conclude that GA requires more matrix metalloproteinases than sarcoidosis. Moreover, we also found that MMP-9 staining in elastic fibers showed the best efficiency in distinguishing GA versus sarcoidosis as predicted by the model. The GA samples in our study showed two histological patterns, palisading and interstitial. Whether these two patterns represent separate histological types or an order of evolution remains to be determined [17]. There also exist cases in which the granulomatous features are not fully developed as the “interstitial” variant [5]. We found that patients with palisading histiocytic infiltration have a longer disease course than those with the interstitial pattern, while the latter showed stronger levels of MMP-9 expression. Therefore, we inferred that palisading granuloma is the mature type of GA, and that MMP-9 may be involved in the chemotaxis and migration of histiocytes in the early stages of GA. Inflammatory responses comprise a series of cellular responses, macrophages, and other effector cells, which then produce a group of matrix metalloproteinases with different functions that respond to environmental stimuli (trauma, host response, and tissue repair) [26]. As a result of these processes, we propose that the surrounded unidentified antigen may induce histiocytes to produce other effector MMPs, such as MMP-1 or MMP-3 [27, 28], to degrade collagen fibers in the later stages of GA when histiocytes are showing palisading infiltration.

Sarcoidosis is a multisystem granulomatous disorder whose pathogenesis involves macrophages that present the internalized antigen to lymphocytes, activate lymphocytes and macrophages (initiation phase) and migrate (accumulation phase), and further release inflammatory mediators to recruit more histiocytes to the granuloma center to expand the Th1 response (effector phase). After the effector phase, granulomas tend to then involve Th2 cell-dominated resolutions or TGF-*β* mediated fibrosis (resolution phase) [11]. Results from previous studies have also shown that MMP-9 can act on nonmatrix proteins, such as cytokines and chemokines, and, in this way, affect the activity of these proteins [26, 29]. We have found that in lesion samples of sarcoidosis with abundant lymphocytes, the histiocytes expressed less MMP-9, while in granulomas surrounded by peripheral fibrous tissue, fewer lymphocytes were observed and the expression of MMP-9 was stronger. It is inferred that MMP-9 may play role mainly in the resolution stage, promoting the fibrosis of granulomas by inducing the production of molecules such as TGF-*β* [30], or play a role by regulating the function of cytokines and chemokines, effects which are not observed in the accumulation and effector stages.

Granulomas develop when the immune system attempts to block substances that are insoluble and cannot be eliminated to prevent them from spreading to other parts of the body [17]. In GA, the JAK-STAT pathway responds to IFN-*γ* to mediate the crosstalk between lymphocytes and histiocytes [15]. The results from our study demonstrate that as the degradation area of collagen fibers in GA is increasing, there is a corresponding increase in pSTAT1 expression within the nuclei of histiocytes. One possible scenario is that central necrotic substances cause proinflammatory responses and the induction of macrophages to differentiate into M1 effector cells expressing tumor necrosis factor-*α* (TNF-*α*), MMP-2, and MMP-9 under the influence of pSTAT1 and other molecules [31, 32], resulting in matrix degradation. Alternatively, histiocytes expressing pSTAT1 could recruit more lymphocytes and histiocytes by releasing cytokines and chemokines to enclose the central degradation area and thus limit the expansion of inflammation.

In the early stages of sarcoidosis, Th1 immune responses lead to M1 macrophage assembly and moderate lymphocyte infiltration surrounding the granuloma. However, these lymphocytes dissipate in the mature lesion as M1 macrophages are transformed into the M2 type [17]. In our study, we found that, in sarcoidosis with substantial lymphocyte infiltration, pSTAT1 showed weak staining in histiocytes; however, as the disease progressed, lymphocyte infiltration gradually decreased while the intensity of pSTAT1 staining increased. One possible explanation for these findings is that histiocytes in the early stages of inflammation are mainly responsible for internalizing and presenting antigens, along with expressing costimulatory molecules to activate lymphocytes. In the accumulation stage, histiocytes mainly express chemokines and receptors that respond to the recruitment of lymphocytes. At this stage of the disease, the expression of JAK-STAT is not strong. In the effector stage, histiocytes express IL-12 and IL-18 cytokines, as activated by pSTAT1 and other transcription factors, to further amplify Th1 responses which then promote granuloma maturation. It has been reported that the JAK-STAT pathway is involved in the regulation of Th1/Th2 responses in tuberculosis as well as in promoting the expression of Th2-related cytokines under the action of long, noncoding RNAs, to mediate antigen dissolution [33]. Here, we found that in sarcoidosis, pSTAT1 was positively correlated with MMP-9, suggesting that pSTAT1 may promote granulomatous fibrosis by inducing the expression of MMP-9 after the effector phase.

The limitations of this study include the small sample size, and the focus was mainly directed to the comparison between these two diseases to investigate the different expression of MMP-9 and pSTAT1. Future studies will be required to provide further insights into the process of granuloma formation and thus the foundation for the development of discriminatory diagnoses and treatments.

## 5. Conclusions

In summary, MMP-9 and pSTAT1 showed differential expressions in GA and sarcoidosis, especially with regard to MMP-9 in elastic fibers. In GA, we propose that MMP-9 may play roles in the early stages of the disease through inducing histiocytes migration, while pSTAT1 is involved in the central degeneration in palisading GA. In sarcoidosis, it appears that pSTAT1 may promote granuloma maturation at the effector stage, while MMP-9 may be involved with promoting granuloma fibrosis.

## Figures and Tables

**Table 1:** Comparison of clinical and histological characteristics of patients with GA and sarcoidosis.

	Granuloma annulare		Sarcoidosis		
	(*n* = 36)		(*n* = 26)		*P* value^∗^
Male sex	23	(88.5)	9	(34.6)	**0.023**
Age at diagnosis, years	47	(14.4-53.9)	53	(38.6–62.2)	**0.019**
Age category, years					**0.029**
<20	10	(27.8)	1	(3.9)	
20–50	8	(22.2)	9	(34.6)	
≥50	18	(50.0)	16	(61.5)	
Duration, years	2	(0.8–3)	0.6	(0.2–2)	0.058
Duration category, years					0.058
<1	9	(25.0)	14	(53.9)	
1–3	13	(36.1)	7	(26.9)	
≥3	14	(38.9)	5	(19.2)	
Involved sites^a^					
Head	7	(19.4)	14	(53.8)	
Trunk	9	(25)	10	(38.5)	
Upper limbs	15	(41.7)	14	(53.8)	
Lower limbs	12	(33.3)	13	(50.0)	
Hands	12	(33.3)	7	(26.9)	
Feet	7	(19.4)	6	(23.1)	
Distribution					0.074
Localized	35	(97.2)	21	(80.8)	
Generalized	1	(2.8)	5	(19.2)	
Pulmonary involvement	0		4	(15.4)	0.056
Diabetes	4	(11.1)	4	(15.4)	0.71
Hypertension	7	(19.4)	5	(19.2)	1.00
Depth of granulomatous infiltration, *μ*m	1360	(1020–2280)	3140	(1710–6645)	**<0.001**
Area of granuloma, mm^2^	2.5	(1.3–5.7)	6.5	(2.1–15.2)	**0.005**
Histiocytes density^b^	677.8	(510.7–1044.6)	718.9	(522.7–913.1)	0.38
Lymphocytes density^b^	228.5	(126.6–439.6)	257.1	(155.7–536.5)	0.45
Multinucleated giant cells density^b^	0	(0–1.3)	0.3	(0–1.1)	0.95
Pattern of histiocytic infiltration					
Palisading	13	(36.1)			
Interstitial	23	(63.9)			

GA: granuloma annulare. Data are presented as *n* (%) or median (interquartile range). ^a^Patients might be included in more than one category. ^b^The number of cells in the granuloma category area/(the number of pixels in the granuloma category area/1,000,000). ^∗^Statistically significant *P* values are shown in bold.

## Data Availability

The cell segmentation and score data, as well as the clinic information used to support the findings of our study are available from the corresponding author upon request.

## Supplementary Materials

Supplementary Materials The histopathologic features in granuloma annulare and sarcoidosis (Supplemental Figure 1) revealed from the whole scan sections and quantification of immunohistochemical staining for MMP-9 and pSTAT1 in different subsets of granuloma annulare and sarcoidosis patients (Supplemental Figure 2) are available in the supplementary materials.

## References

[1] Joshi T. P., Duvic M. (2022). Granuloma annulare: an updated review of epidemiology, pathogenesis, and treatment options. *American Journal of Clinical Dermatology*.

[2] Haimovic A., Sanchez M., Judson M. A., Prystowsky S. (2012). Sarcoidosis: a comprehensive review and update for the dermatologist: part i. Cutaneous disease. *Journal of the American Academy of Dermatology*.

[3] Ali M. M., Atwan A. A., Gonzalez M. L. (2010). Cutaneous sarcoidosis: updates in the pathogenesis. *Journal of the European Academy of Dermatology and Venereology : JEADV*.

[4] Piette E. W., Rosenbach M. (2016). Granuloma annulare: clinical and histologic variants, epidemiology, and genetics. *Journal of the American Academy of Dermatology*.

[5] Ronen S., Rothschild M., Suster S. (2019). The interstitial variant of granuloma annulare: clinicopathologic study of 69 cases with a comparison with conventional granuloma annulare. *Journal of Cutaneous Pathology*.

[6] Cardoso J. C., Cravo M., Reis J. P., Tellechea O. (2009). Cutaneous sarcoidosis: a histopathological study. *Journal of the European Academy of Dermatology and Venereology : JEADV*.

[7] Cohen P. R., Carlos C. A. (2015). Granuloma annulare mimicking sarcoidosis: report of patient with localized granuloma annulare whose skin lesions show 3 clinical morphologies and 2 histology patterns. *The American Journal of Dermatopathology*.

[8] Shibayama A., Sugita K., Narukawa K. (2017). Granuloma annulare can occur on a scar, mimicking sarcoidosis. *Clinical and Experimental Dermatology*.

[9] Takama H., Ohshima Y., Ando Y. (2020). Annular sarcoidosis with geographic appearance in a patient with systemic sarcoidosis. *Acta Dermato-Venereologica*.

[10] Volkman H. E., Pozos T. C., Zheng J., Davis J. M., Rawls J. F., Ramakrishnan L. (2010). Tuberculous granuloma induction via interaction of a bacterial secreted protein with host epithelium. *Science*.

[11] Iannuzzi M. C., Fontana J. R. (2011). Sarcoidosis. *JAMA*.

[12] Taylor J. L., Hattle J. M., Dreitz S. A. (2006). Role for matrix metalloproteinase 9 in granuloma formation during pulmonary mycobacterium tuberculosis infection. *Infection and Immunity*.

[13] Malur A., Mohan A., Barrington R. A. (2019). Peroxisome proliferator-activated receptor-*γ* deficiency exacerbates fibrotic response to mycobacteria peptide in murine sarcoidosis model. *American Journal of Respiratory Cell and Molecular Biology*.

[14] Damsky W., Thakral D., Mcgeary M. K., Leventhal J., Galan A., King B. (2020). Janus kinase inhibition induces disease remission in cutaneous sarcoidosis and granuloma annulare. *Journal of the American Academy of Dermatology*.

[15] Chu C. Y. (2021). New targets in treating granuloma annulare. *The Journal of Allergy and Clinical Immunology*.

[16] Wang A., Singh K., Ibrahim W., King B., Damsky W. (2020). The promise of JAK inhibitors for treatment of sarcoidosis and other inflammatory disorders with macrophage activation: a review of the literature. *The Yale Journal of Biology and Medicine*.

[17] Asai J. (2017). What is new in the histogenesis of granulomatous skin diseases?. *The Journal of Dermatology*.

[18] Zhao C. C., Han Q. J., Ying H. Y. (2022). Tnfsf15 facilitates differentiation and polarization of macrophages toward m1 phenotype to inhibit tumor growth. *Oncoimmunology*.

[19] Rosenbaum J. T., Pasadhika S., Crouser E. D. (2009). Hypothesis: sarcoidosis is a stat1-mediated disease. *Clinical Immunology (Orlando, Fla.)*.

[20] Zhou T., Casanova N., Pouladi N. (2017). Identification of JAK-STAT signaling involvement in sarcoidosis severity via a novel microRNA-regulated peripheral blood mononuclear cell gene signature. *Scientific Reports*.

[21] Song M., Xing X. (2023). miR-6742-5p regulates the invasion and migration of lung adenocarcinoma cells via mediating FGF8/ERK12/MMP9/MMP2 signaling pathway. *Aging*.

[22] Dopytalska K., Gabzdyl N., Szczerba M., Szymańska E., Walecka I. (2022). Is biologic therapy the future of granuloma annulare treatment?. *Dermatologic Therapy*.

[23] Annunziato F., Romagnani C., Romagnani S. (2015). The 3 major types of innate and adaptive cell-mediated effector immunity. *The Journal of Allergy and Clinical Immunology*.

[24] Lynch J. M., Barrett T. L. (2004). Collagenolytic (necrobiotic) granulomas: part 1--the "blue" granulomas. *Journal of Cutaneous Pathology*.

[25] Salgame P. (2011). Mmps in tuberculosis: granuloma creators and tissue destroyers. *The Journal of Clinical Investigation*.

[26] Parks W. C., Wilson C. L., López-Boado Y. S. (2004). Matrix metalloproteinases as modulators of inflammation and innate immunity. *Nature Reviews. Immunology*.

[27] Budden T., Gaudy-Marqueste C., Porter A. (2021). Ultraviolet light-induced collagen degradation inhibits melanoma invasion. *Nature Communications*.

[28] Mirastschijski U., Lupše B., Maedler K. (2019). Matrix metalloproteinase-3 is key effector of TNF-*α*-induced collagen degradation in skin. *International Journal of Molecular Sciences*.

[29] Xu T., Gao S., Liu J., Huang Y., Chen K., Zhang X. (2021). MMP9 and IGFBP1 regulate tumor immune and drive tumor progression in clear cell renal cell carcinoma. *Journal of Cancer*.

[30] Youn M., Huang H., Chen C. (2019). MMP9 inhibition increases erythropoiesis in RPS14-deficient del(5q) MDS models through suppression of TGF-*β* pathways. *Blood Advances*.

[31] Barros M. H., Hauck F., Dreyer J. H., Kempkes B., Niedobitek G. (2013). Macrophage polarisation: an immunohistochemical approach for identifying M1 and M2 macrophages. *PLoS One*.

[32] Fayyazi A., Schweyer S., Eichmeyer B. (2000). Expression of IFNgamma, coexpression of TNFalpha and matrix metalloproteinases and apoptosis of T lymphocytes and macrophages in granuloma annulare. *Archives of Dermatological Research*.

[33] Jianfang W., Hui W., Le K. (2022). LINC00870 regulates Th1/Th2 via the JAK/STAT pathway in peripheral blood mononuclear cells infected with Mycobacterium tuberculosis. *International Immunopharmacology*.

