# Supplementary material for: Immunohistochemical Features of MMP-9 and pSTAT1 in Granuloma Annulare and Sarcoidosis: A Comparative Study of 62 Cases
Source: J Immunol Res. 2023 Mar 14;2023:4098459. doi: 10.1155/2023/4098459 (PMC10030222; doi:10.1155/2023/4098459)

Supplementary information

Supplementary Figure Legend
Supplementary Figure 1
Supplementary Figure 2

Supplementary Figure Legend

Supplemental figure 1. Histopathologic features in granuloma annulare (GA) and sarcoidosis. (A, B) Representative hematoxylin and eosin (H&E) staining images in patients with GA (A) and sarcoidosis (B), the line segment measures the infiltration depth. C, Quantification of granulomatous infiltration depth and granuloma area in GA and sarcoidosis. (D, E) Representative H&E staining images in biopsies with palisading (D) and interstitial GA (E), solid lines circle the granuloma, dotted lines circle the mucinous collagen degradation. F, Quantification of granuloma area and duration in patients with palisading and interstitial GA. (G, H) Representative H&E staining images in sarcoidosis patients with nodular (G) and non-nodular (H) manifestation, the line segment measures the infiltration depth, arrow heads indicate the multinucleated giant cells (MNGCs). I, Quantification of granulomatous infiltration depth and MNGCs density in sarcoidosis patients with nodular and non-nodular manifestation. Scale bar = 1 mm in all images.

Supplemental figure 2. Quantification of immunohistochemical (IHC) staining for MMP-9 and pSTAT1 in different subsets of granuloma annulare and sarcoidosis patients. A, Quantification of IHC staining for MMP-9. B, Quantification of IHC staining for pSTAT1.

Supplementary Figure 1

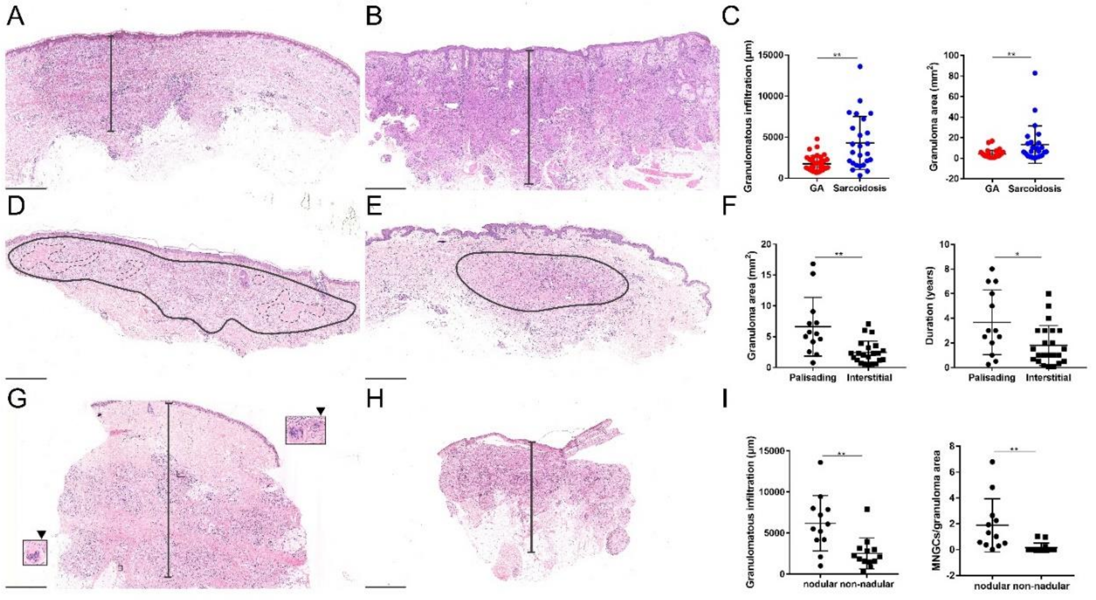

Supplementary Figure 2

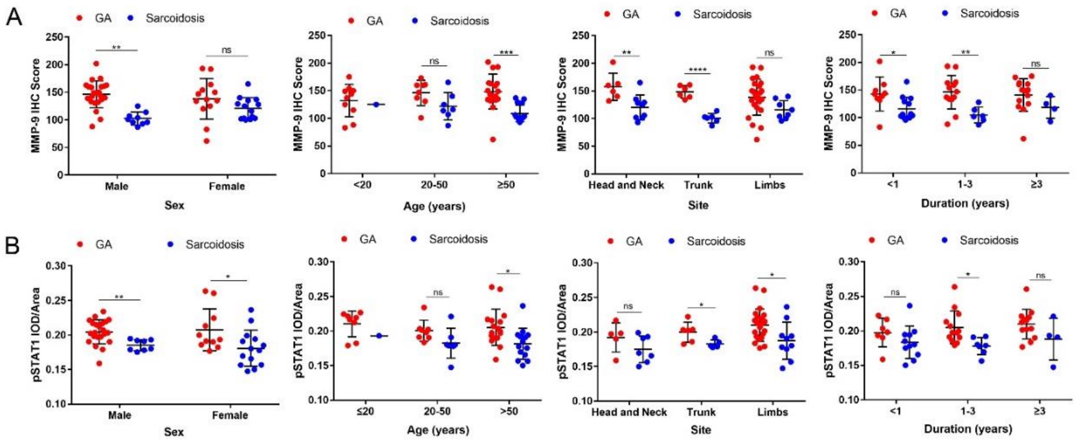

Supplement: Supplementary Materials — The histopathologic features in granuloma annulare and sarcoidosis (Supplemental Figure 1) revealed from the whole scan sections and quantification of immunohistochemical staining for MMP-9 and pSTAT1 in different subsets of granuloma annulare and sarcoidosis patients (Supplemental Figure 2) are available in the supplementary materials. [file 4098459.f1.pdf]
